# Does Environmental Education Always Contribute to Remanufacturing Supply Chain Development?

**DOI:** 10.3390/ijerph20064725

**Published:** 2023-03-07

**Authors:** Chunmei Li, Tianjian Yang, Zijing Bian

**Affiliations:** 1 School of Economics and Management, Beijing University of Posts and Telecommunications, Beijing 100876, China; 2 School of Modern Post (School of Automation), Beijing University of Posts and Telecommunications, Beijing 100876, China; 3 Department of Economic Theory Management, College of Social Sciences and Humanities, Moscow State Normal University, 119991 Moscow, Russia

**Keywords:** environmental education, sustainable development, remanufacturing, yield uncertainty, game theory

## Abstract

Remanufacturing, as an effective way to save resources and alleviate environmental pollution, has gradually become a sustainable practice. Environmental education contributes to the development of remanufacturing by increasing the number of consumers willing to purchase remanufactured products (RPs). However, the incumbent manufacturer usually has limited remanufacturing capability together with yield uncertainty, making a third-party remanufacturer (3PR) an alternate channel choice. This study develops an analytical model to examine the effects of environmental education on a retailer’s choice of remanufacturing channels under in-store competition. Results show that consumer environmental education has the potential to significantly improve the retailer and supply chain profits, and temperate environmental education is always desirable for 3PR. The introduction of 3PR benefits the consumer when the retailer’s remanufacturing technology level is low. Furthermore, when the environmental impact of defective RPs is relatively high, and environmental education is temperate, selecting a 3PR will enhance environmental sustainability. This study also shows that 3PR can help achieve a win–win situation when environmental education and consumer acceptance of RPs are both in a certain range.

## 1. Introduction

In recent years, social and economic developments have increased the demands placed upon the enterprise’s production to protect the environment. There were 53.6 Mt of electronic waste reported globally in 2019, of which 82.6% had an unknown fate [1]. In 2020, waste electrical and electronic equipment accounted for more than USD 57 billion in the global market [2]. A large amount of waste will have a significant impact on the economy and the environment. There is a probability that some of the waste can be recovered and reused through remanufacturing. In addition, compared to new products, a company can save 40–65 percent in manufacturing costs by remanufacturing [3]. For example, the remanufacturing of HP and EPSON achieved a cost saving of 65% [4]. To reduce the negative environmental impact, a growing number of enterprises began to produce remanufactured products. Remanufacturing has gradually become a sustainable practice in the supply chain.

However, only a small percentage of consumers who have a systematic knowledge of remanufactured products are willing to choose remanufactured products [5]. Research holds that environmental education plays an important role in shaping attitudes about sustainable consumption [6], and one of the key causal barriers to smart waste management is the lack of environmental education [7]. It is urgent for governments to improve potential consumers’ product knowledge and trust to improve remanufactured products’ purchasing intentions [8]. Therefore, governments should invest in education to reduce energy consumption and protect the environment [9]. Consumer environmental education has an important role in changing the mindset of consumers. For example, through environmental education, consumers can learn about the advantages of RPs, such as saving manufacturing costs, recycling used products, lowering prices, etc., which is conducive to increasing consumer awareness of RPs. Consumers who have been provided with environmental education are unbiased between RPs and NPs. In this paper, we consider that environmentally educated consumers only care about whether the function of RPs is the same as new products, and this type of consumers can be called functionality-oriented consumers (FOCs). We propose that the more effective the environmental education, the higher the market share of FOCs.

Furthermore, there are many challenges associated with information uncertainty in the supply chain [10], such as yield uncertainty. In general, the incumbent remanufacturer usually has limited remanufacturing capability together with yield uncertainty issues, while some power remanufacturers have very stable remanufacturing capabilities. A remanufacturer may be a retailer who resells new products and manufactures remanufactured products. For example, an auto parts retailer in Guangzhou, Oruid Automobile Engine Technology, produces and sells remanufactured engines as well as new Mercedes Benz, BMW, and Audi parts [11]. For in-house remanufacturing, yield uncertainty will significantly impact the remanufactured product qualification rate. Thus, the retailer, such as Oruid Automobile Engine Technology, can decide to whether to introduce a power remanufacturer to solve yield uncertainty from in-house remanufacturing. This study considers a retailer who sells the remanufactured products and can decide whether to perform in-house remanufacturing or purchase from a third-party remanufacturer (3PR). The 3PR usually has a strong remanufacturing production technology, and the introduction of 3PR can eliminate yield uncertainty in the remanufacturing processes. Thus, there are some questions: Does environmental education always facilitate the remanufacturing supply chain development? How do environmental education and yield uncertainty affect the remanufacturing channel selections? Is environmental education always beneficial for supply chain profit, consumer surplus, and environmental impact?

To solve the above problem, we examine a remanufacturing supply chain comprising a new product manufacturer, a retailer, and a third-party remanufacturer (denoted by 3PR). The remanufacturing market is divided into two types: functionality-oriented consumers (FOCs) and newness-conscious consumers (NCCs). NCCs who focus on the novelty of products only buy NPs regardless of the price discount of RPs. There are two production patterns in the retailer’s remanufactured products (RPs): in-house remanufacturing (Model IR) and outsourced manufacturing (Model OR). When the retailer chooses in-house remanufacturing, there exists yield uncertainty, while the 3PR does not.

The main findings of this study are as follows. First, wholesale prices are decreasing in environmental education while the order quantities are increasing as the size of the FOC segment enlarges. Environmental education has the potential to significantly improve consumer surplus, the retailer, and supply chain profits. Second, the manufacturer and retailer will achieve a win–win situation through outsourcing remanufacturing to the third-party remanufacturer when environmental education and consumer acceptance for the remanufactured product are both in a certain range. The 3PR’s profit will be the highest when both consumer acceptance for RPs and environmental education are in a certain range. Finally, outsourcing remanufacturing benefits the consumer when the retailer’s remanufacturing technology level is low. Relatively higher environmental impacts of defective RPs and temperate environmental education can enhance the environmental sustainability of outsourcing remanufacturing.

The remainder of this paper is organized as follows. The related literature of this paper is presented in Section 2. Section 3. formulates the game model and describes the assumptions. Section 4. analyzes the equilibrium outcomes. Section 5. analyzes the supply chain profit, consumer surplus, and environmental performance. Section 6. concludes the main findings and proposes future research directions.

## 2. Literature Review

Our research mainly relates to the literature on remanufactured products, yield uncertainty, and environmental education.

### 2.1. Remanufactured Products

One stream of the literature investigates the marketing issue of remanufactured products. For example, Alyahya et al. [12] developed an integrated model based on complexity theory to study factors that influence consumer decisions to purchase remanufactured products. Dobbelstein and Lochner [13] examined the factors influencing purchase intentions for recycled products, including differences between Germans and South Africans. Alqahtani and Gupta [14] pointed out that offering warranties on remanufactured products can minimize the costs incurred by remanufacturers and increase consumer confidence in remanufactured products. Yan et al. [15] investigated the channel structures for marketing remanufactured products and found that in comparison with subcontracting, the more retailers in the market, the more environmentally friendly the e-channel is. Xue et al. [16] studied the strategic aspects of competition in and from remanufacturing between a high-end firm and a low-end firm. Alegoz [17] examined the effects of an actor entering the remanufacturing industry if there is already an actor remanufacturing by comparing three remanufacturing systems. Xia et al. [18] pointed out that product-oriented product service systems benefit from the increased profit for the original equipment manufacturer and third-party remanufacturer. From the third-party remanufacturing’s perspective, Jin et al. [19] pointed out that third-party remanufacturing could be less detrimental than the OEM’s in-house remanufacturing. From the view of cost information sharing, Huang et al. [20] investigated the incentives of cost information sharing between the third-party remanufacturer and the original equipment manufacturer. Baghdadi et al. [21] explored a tradeoff between customers’ expected costs and dealers’ expected profit when a two-dimensional warranty and post-warranty service are provided with remanufactured products. Qiao and Su [22] studied the optimal prices and quality of new and remanufactured products by segmenting the market as the indifferent segment and the new product-only segment. Nevertheless, a significant difference between their work and ours is that our study focuses on environmental education in the remanufacturing supply chain with yield uncertainty, and examines how environmental education affects the decision on remanufacturing production channels.

### 2.2. Yield Uncertainty

Our work is also related to the literature on yield uncertainty. For example, Hsieh and Lai [23] examined the supply chain members’ decisions in the context of production uncertainty in producing high-quality products. Hsu and Bassok [24] developed a two-stage stochastic program to determine if the optimal production input and allocation under demands and yields are random. Metzker et al. [25] demonstrated that the robust optimization methodology immunizes the system against yield uncertainty. Based on first-order optimality conditions, Freeman et al. [26] studied the optimal use of downward substitution for a capacitated manufacturer facing uncertain supply in a single-period (multi-period) setting and stochastic programming techniques. Peng et al. [27] examined how yield uncertainty affects the suppliers’ entry decisions and the retailer’s profit and consumer surplus. Sharma et al. [28] presented an integrated approach for determining the optimal biofuel supply chain by taking biomass yield uncertainty into account. Cai et al. [29] investigated the optimal input quantity decisions in a vendor-managed inventory supply chain considering demand and yield uncertainty. Cai et al. [30] further investigated the impact of supply capability and strategic customer behavior on the supply chain when facing strategic customers under demand and yield uncertainty. Kouvelis and Li [31] studied an offshore outsourcing strategy for a buyer of a produced good under supply yield uncertainty. When faced with an unreliable and capital-constrained supplier with random yields, Yuan et al. [32] explored how the manufacturer makes optimal sourcing decisions. Talay and Ozdemir-Akyildirim [33] examined how a producer inputs purchases and allocates semi-processed items in a multi-product, two-stage production process under conditions of yield uncertainty. Shao et al. [34] examined how yield uncertainty affects the equilibrium outcome in a hybrid market with spot and forward transactions. Lowe et al. [35] proposed an optimized production planning approach under yield uncertainty in semiconductor manufacturing. More and more studies have focused on yield uncertainty in remanufacturing supply chains. For example, Niu et al. [11] explored the retailer’s remanufactured channels consisting of in-house remanufacturing with yield uncertainty and external remanufacturing from the third-party remanufacturer. Liao et al. [36] found that producing new products at a higher cost may be more profitable than remanufacturing, and the reactive strategy can lessen the risk of stock-out when remanufacturability is uncertain. Zhu et al. [37] examined the effects of yield uncertainty and market competition on remanufacturing decisions under three classical market scenarios. Differently, we examine yield uncertainty relating to remanufacturability considering the consumers’ environmental education, which has been addressed in few previous studies.

### 2.3. Environmental Education

Another related stream of literature is on the topic of environmental education. Most research on environmental education has focused on empirical and experimental aspects. For example, Begum et al. [38] pointed out that environmental education strategies that are effective include proactive environmental education, environmental awareness, and real-life simulations. Yang et al. [39] examined how narrative-based environmental education affects children’s awareness of the environment. Using public participation as a mediator, Niu et al. [40] empirically analyzed the impact of environmental education and publicity on environmental governance performance. Some literature pointed out that environmental education has a positive impact on human behavior. For instance, van de Wetering et al. [41] showed that environmental education has the potential to improve students’ environmental knowledge, attitudes, intentions, and behaviors. Steils [42] examined how in-store customer education contributes to interrupting impulsive consumption of unhealthy foods using a survey and an experimental study design. With the inclusion of the nature-in-self (INS) scale, Lieflander et al. [43] examined how well environmental education programs promote connectedness with nature. Some scholars have studied the role of environmental education in the supply chain. For example, Agrawal et al. [44] found that environmental education for consumers can improve the relative environmental performance of leasing under some conditions. Merkuryev et al. [45] discussed the use of simulation-based business games for supply chain management training and education. Zhong et al. [46] examined the effects of consumer green education on the recycler’s channel choice of Agency channel or Self-Run channel and found that moderate consumer green education benefits a new product seller from the remanufactured goods’ cannibalization in the Agency channel. Zhou et al. [47] explored how consumer education affects the interaction within the remanufacturing supply chain. Wang et al. [48] investigated the effect of consumer education on the reverse channel designs considering different collection and remanufacturing capabilities. Then, Wang et al. [49] further investigated the impact of warranty service and consumer education on remanufacturing decisions of an original equipment manufacturer. Unlike previous literature, we consider consumer environmental education in remanufacturing supply chain, as well as considering yield uncertainty when the retailer conducts the in-house remanufacturing. Furthermore, this study also explores the impact of environmental education on the remanufactured products’ production channels.

## 3. Model

**Figure 1:**
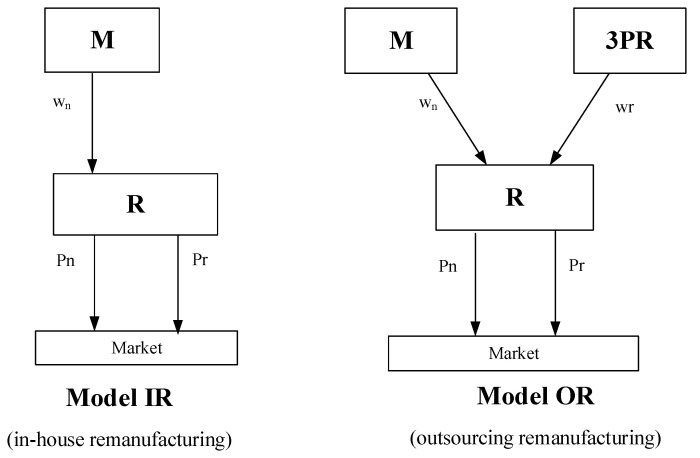
Supply chain structures.

**Figure 2:**
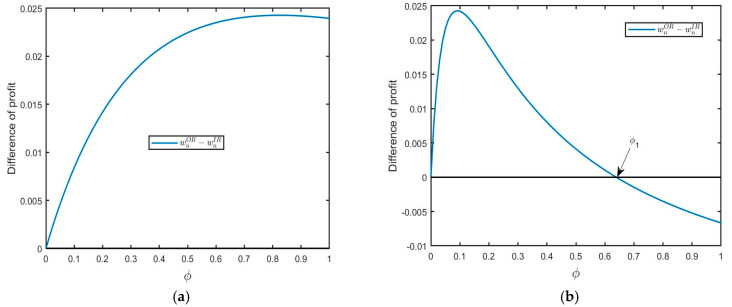
The impact of ϕ on difference: (**a**) $\delta <1-3\sigma^{2}/\mu^{2}\;(\mu =0.9,\sigma =0.2,\delta =0.5)$ and (**b**) $\delta <1-3\sigma^{2}/\mu^{2}\;(\mu =0.9,\sigma =0.2,\delta =0.9)$.

To investigate how consumer environmental education affects the retailer’s remanufactured products’ production pattern and the sustainability of the supply chain, we assume there are a new product manufacturer (denoted by M) and a retailer (denoted by R) in the market. The retailer procures the new products (NPs) from the new product manufacturer at a unit wholesale price and also sells the remanufactured products. There are two production patterns in the retailer’s remanufactured products: in-house remanufacturing and outsourced manufacturing. (1) The retailer produces the remanufactured products (in-house remanufacturing, Model IR, Figure 1); (2) the retailer purchases from a third-party remanufacturer (denoted by 3PR) at a unit wholesale price (outsourcing remanufacturing, Model OR, Figure 1).

In model IR, in-house remanufacturing has issues related to yield uncertainty, which can result in some defective remanufactured products. For a given production quantity q, the actual output is εq, where ε∈(0,1) denote the yield uncertainty with mean μ and variance $\sigma^{2}$. Note that $\mu >\mu^{2}+\sigma^{2}$ and 0<σ<1/2 (Note that $Var(\mu )=E(\mu^{2})-{\left(E(\mu )\right)}^{2}$. 0<μ<1, thus $E(\mu^{2})<E(\mu )$. Therefore, we can obtain $Var(\mu )<E(\mu )-{\left(E(\mu )\right)}^{2}=\mu -\mu^{2}$, that is $\sigma^{2}<\mu -\mu^{2}=-{\left(1-\mu \right)}^{2}+\frac{1}{4}$. Hence, we have Var(μ)<1/4. This indicates that σ is smaller than 1/2.). For outsourcing remanufacturing, the 3PR is equipped with more advanced technologies for remanufacturing, improving its reliability in remanufacturing. As a result, it is possible for the 3PR to avoid yield uncertainty by ensuring the quality of all the delivered products. Without loss of generality, the marginal production cost of the new products and remanufactured products are assumed to be constant, and both normalized to zero. The notations in this paper are summarized in Table 1.

We assume all consumers are divided into two types: functionality-oriented consumers (FOCs) and newness-conscious consumers (NCCs). FOCs who have been provided with environmental education only care about whether the function of RPs is the same as that of NPs. On the other hand, NCCs who focus on the novelty of products only buy NPs regardless of the price discount of RPs. The market potential is normalized to 1. The proportion of the FOCs segment is ϕ∈(0,1), while the proportion of the NCCs only segment is 1−ϕ. Let $p_{i},q_{i}$ denote the production quantity and retail price in this paper and i=n,r represent the NPs and RPs, respectively. We assume consumers’ willingness-to-pay for NPs is v, which is heterogeneous and uniformly distributed over [0, 1]. Each FOC’s willingness-to-pay for RPs is a fraction δ∈(0,1) of NPs, and all NCCs’ willingness-to-pay for RPs is 0. The net utility of NCCs from NPs is $v-p_{n}$; the net utility of FOCs from NPs and RPs are $v-p_{n}$ and $\delta v-p_{r}$, respectively. Following Zhou et al. [47], we can obtain the inverse demand functions of NPs and RPs as follows:

(1) $$p_{n}=1-q_{n}-\delta q_{r}$$

(2) $$\;p_{r}=\delta \left(1-q_{n}-\delta q_{r}\right)-\frac{\delta \left(1-\delta \right)q_{r}}{\phi }\;$$

In model IR, the actual output of the remanufactured product is $\varepsilon q_{r}^{IR}$. The profit functions of the new product supplier and the retailer are as follows:

(3) $$\;\pi_{M}^{IR}=w_{n}^{IR}q_{n}^{IR}\;$$

(4) $$\;\pi_{R}^{IR}=\left(p_{n}^{IR}-w_{n}^{IR}\right)q_{n}^{IR}+p_{r}^{IR}\varepsilon q_{r}^{IR}$$

In model OR, the actual output of the remanufactured product is $q_{r}^{OR}$. The profit functions of the new product supplier and the retailer are as follows:

(5) $$\pi_{M}^{OR}=w_{n}^{OR}q_{n}^{OR}$$

(6) $$\pi_{3PR}^{OR}=w_{r}^{OR}q_{r}^{OR}$$

(7) $$\pi_{R}^{OR}=\left(p_{n}^{OR}-w_{n}^{OR}\right)q_{n}^{OR}+\left(p_{r}^{OR}-w_{r}^{OR}\right)q_{r}^{OR}$$

The sequence of events is as follows. In stage 1, the retailer decides which production model to introduce, in-house remanufacturing or outsourcing remanufacturing from a 3PR. In stage 2, the manufacturer and the 3PR (model OR) determine the wholesale prices. In stage 3, the retailer decides the order quantities of NPs and RPs. Finally, according to their individual utility, consumers purchase either NPs or RPs at retail prices.

## 4. Analysis

In this section, using backward induction to solve the games, the equilibrium outcomes are summarized in Table 2. For ease of simplified calculation and exposition, we define some items in Table 3. The derivation and proof of this paper are in Appendix A.

### 4.1. Wholesale Price

**Lemma**  **1.** *The wholesale prices of NPs and RPs are decreasing in *ϕ, *that is* $\frac{\partial w_{n}^{IR}}{\partial \phi }<0$*,* $\frac{\partial w_{n}^{OR}}{\partial \phi }<0$  *and*  $\frac{\partial w_{r}^{OR}}{\partial \phi }<0$.

Lemma 1 shows that the wholesale prices of NPs and RPs both decrease in ϕ. When the size of the FOCs segment enlarges, the new product manufacturer has the incentive to decrease the wholesale price in order to stimulate the orders of NPs in both model IR and OR. For RPs, as the FOCs segment expands, the competition between 3PR and the new product manufacturer intensifies, which drives the 3PR to decrease the wholesale price.

**Lemma**  **2.** *The wholesale prices of NPs in model OR is higher than in model IR (i.e.,* $w_{n}^{OR}-w_{n}^{IR}>0$*) if* $3\sigma^{2}<\mu^{2}$, $\delta <1-3\sigma^{2}/\mu^{2}$  *or* $\delta >1-3\sigma^{2}/\mu^{2}$  *and* $\phi <\phi_{1}$*. Otherwise, we have * $w_{n}^{OR}-w_{n}^{IR}<0$.

Lemma 2 demonstrates that the wholesale prices of NPs in model OR are higher than that in model IR when both the consumer acceptance for the RPs and yield uncertainty are low (see Figure 2a for illustration). In this situation, the new products are more competitive in the market than remanufactured products. Thus, the new product manufacturer has an incentive to raise the wholesale price of NPs. When the consumer acceptance for the RPs is relatively large, and the size of the FOCs segment is relatively small (see Figure 2b for illustration), new products still have a larger market potential than remanufactured products, thereby strengthening its pricing power.

### 4.2. Order Quantity

**Lemma**  **3.** *The order quantities of NPs and RPs are increasing in* ϕ*, that is* $\frac{\partial q_{r}^{OR}}{\partial \phi }>0$, $\frac{\partial q_{r}^{IR}}{\partial \phi }>0$*, and* $\frac{\partial q_{n}^{OR}}{\partial \phi }>0$.

The result in Lemma 3 is unexpected. In theory, it would seem that the NPs market would shrink when the FOCs segment increases. However, a large ϕ also leads to a lower wholesale price for NPs, as illustrated in Lemma 1, which in turn motivates the retailer to purchase more new products. There is no doubt that the retailer will order more RPs as the size of the FOCs segment enlarges. Therefore, both NPs and RPs orders are increasing at the retailer. This phenomenon is termed the sales augment effect by Niu et al. [11].

**Lemma**  **4.** *The order quantity of RPs in model OR is smaller than in model IR (i.e.,* $q_{r}^{OR}-q_{r}^{IR}<0$*). For NPs, the order quantity in model OR is larger than in model IR (i.e.,* $q_{n}^{OR}-q_{n}^{IR}>0$).

According to the equilibrium outcomes, it is easy to obtain that $p_{r}{}^{IR}>p_{r}{}^{OR}-w_{r}$. That is, the retailer’s profit margin of RPs in model OR is reduced, which drives the retailer to order less RPs. Thus, the order quantities of RPs in model OR is smaller than in model IR. For the new products, as the retailer’s profit margin on RPs is reduced in model OR, the retailer will order more new products in order to gain more profit from NPs. Therefore, the order quantities of NPs in model OR is larger than in model IR.

### 4.3. Remanufacturing Choice

**Proposition**  **1.** *The retailer benefits from outsourcing remanufacturing from the 3PR (i.e., *$\pi_{R}^{OR}-E[\pi_{R}^{IR}]>0$*) if* $5\mu^{2}<7\sigma^{2}$*, or,* $5\mu^{2}>7\sigma^{2}$, $\delta >\delta_{3}$  *and* $\phi >\phi_{4}$*; otherwise, we have* $\pi_{R}^{OR}-E[\pi_{R}^{IR}]<0$.

**Figure 3:**
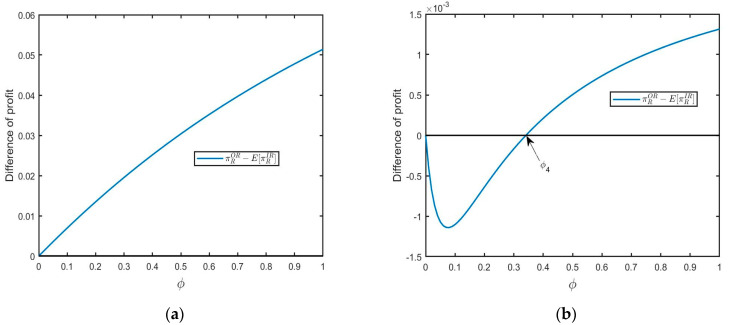
The impact of ϕ on profit difference: (**a**) $5\mu^{2}<7\sigma^{2}(\mu =0.1,\sigma =0.2,\delta =0.5)$ and (**b**) $5\mu^{2}>7\sigma^{2}$ and $\delta >\delta_{3}(\mu =0.5,\sigma =0.2,\delta =0.9)$.

As shown in Figure 3a, when yield uncertainty is relatively large, the retailer has a strong incentive to choose outsourcing remanufacturing from 3PR in order to eliminate yield uncertainty. In situation (a), the retailer can obtain more profits from 3PR. Furthermore, when the yield uncertainty is small, the retailer also tends to purchase from 3PR if both the size of the FOCs segment and consumer acceptance for RPs are larger (see Figure 3b for illustration). In situation (b), remanufactured products are popular in the market as environmental education is effective (ϕ exceeds the threshold value), and consumers have a high acceptance for RPs. Thus, the retailer will benefit from outsourcing remanufacturing.

**Proposition**  **2.** *The new product manufacturer earns a higher profit in model IR (i.e.,*  $\pi_{M}^{OR}-\pi_{M}^{IR}<0$*) if* $\mu^{2}<\sigma^{2}$*, or,* $\mu^{2}>\sigma^{2}$*,*  $\delta >\delta_{1}$  *and* $\phi >\phi_{2}$*; otherwise, we have* $\pi_{M}^{OR}-\pi_{M}^{IR}>0$.

**Figure 4:**
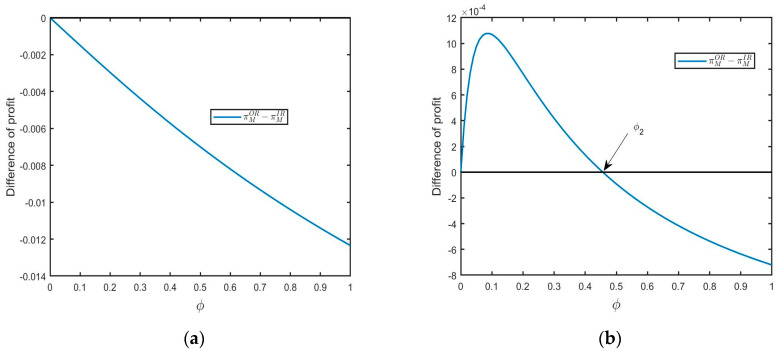
The impact of ϕ on profit difference: (**a**) $\mu^{2}<\sigma^{2}(\mu =0.1,\sigma =0.2,\delta =0.5)$ and (**b**) $\mu^{2}>\sigma^{2}$ and $\delta >\delta_{1}(\mu =0.5,\sigma =0.2,\delta =0.9)$.

Proposition 1 demonstrates the situation in which the retailer is inclined to outsource remanufacturing to a 3PR. Conversely, Proposition 2 shows the situation in which the new product manufacturer prefers in-house remanufacturing. As shown in Proposition 1, when yield uncertainty is relatively large, the retailer is motivated to purchase RPs from a 3PR. For the new product manufacturer, this will benefit the sales stability of new products as the yield uncertainty. Therefore, the new product manufacturer prefers in-house remanufacturing to obtain more profits (see Figure 4a for illustration). When yield uncertainty is relatively small, and consumer acceptance for RPs is relatively large, environmental education will have a significant impact on the new product manufacturer’s choice. The new product manufacturer will gain more profits in model IR when the FOCs segment increases than a threshold value (see Figure 4b for illustration).

**Proposition**  **3.** *The new product manufacturer and retailer will achieve a win–win situation through outsourcing remanufacturing when one of the following conditions holds:*

*(i)*  *if* $5\mu^{2}/7<\sigma^{2}<\mu^{2}$*,* $\delta <\delta_{1}$  *or* $\delta >\delta_{1}$  *and* $\phi <\phi_{2}$.
*(ii)*  *if* $\sigma^{2}<5\mu^{2}/7$*,* $\sigma^{2}<5\mu^{2}/7$*,* $\delta_{3}<\delta <\delta_{1}$  *and* $\phi >\phi_{4}$  *or* $\delta >\delta_{1}$  *and* $\phi_{4}<\phi <\phi_{2}$. *Here,* $\delta_{3}<\delta_{1}$  *and* $\phi_{4}<\phi_{2}$.

**Figure 5:**
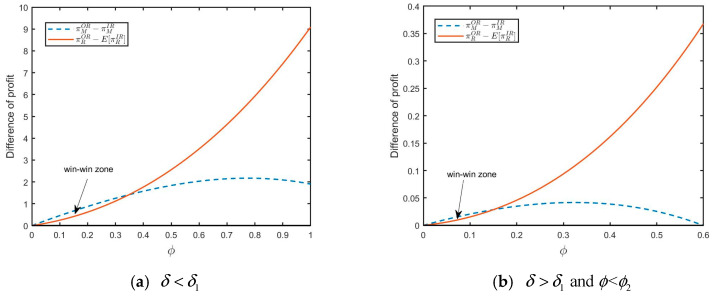
Win–win zone ($\mu^{2}=0.3,\sigma^{2}=0.23$) with (**a**) δ=0.15 and (**b**) δ=0.3.

**Figure 6:**
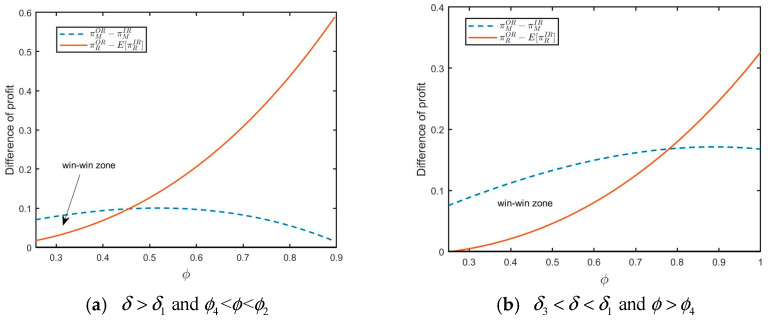
Win–win zone ($\mu^{2}=0.3,\sigma^{2}=0.2$) with (**a**) δ=0.2 and (**b**) δ=0.3.

As pointed out in Proposition 3, the conditions for a win-win situation depend on the level of yield uncertainty, consumer acceptance for RPs, and the size of the FOCs segment. The retailer always prefers outsourcing remanufacturing if $\mu^{2}<7\sigma^{2}/5$ and the new product manufacturer also prefers model OR if $\sigma^{2}<\mu^{2}<17\sigma^{2}/7$, $\delta <\delta_{1}$  or $\delta >\delta_{1}$  and $\phi <\phi_{2}$. Thus, the first win-win situation can be achieved in Proposition 3 (i). When $\sigma^{2}$ is intermediate, consumer acceptance for RPs is relatively small (see Figure 5a for illustration); or consumer acceptance for RPs is relatively large, but the size of the FOCs segment is small (see Figure 5b for illustration). Ultimately, this situation benefits both the retailer and the new product manufacturer. Compared with Figure 5a,b, we find that the win–win zone is getting smaller with consumer acceptance for RPs increases, and the size of the FOCs segment is not large enough. The retailer also tends to purchase RPs if $\mu^{2}>7\sigma^{2}/5$, $\delta >\delta_{3}$ and $\phi >\phi_{4}$. There are also two cases when the new product manufacturer will be inclined to the model OR: (1) if $7\sigma^{2}<7\mu^{2}<17\sigma^{2}$, $\delta <\delta_{1}$ or $\delta >\delta_{1}$ and $\phi <\phi_{2}$, (2) if $\mu^{2}>17\sigma^{2}/7$, $\delta <\delta_{2}$ or $\delta >\delta_{1}$ and $\phi <\phi_{3}$, or $\delta_{2}<\delta <\delta_{1}$ and $\phi >\phi_{3}$, or $\delta >\delta_{1}$ and $\phi_{3}<\phi <\phi_{2}$. As a result, the second win–win situation can be achieved in Proposition 3 (ii). When $\sigma^{2}$ is low (i.e., $\sigma^{2}<5\mu^{2}/7$), consumer acceptance for RPs is intermediate, and the size of the FOCs segment is large (see Figure 6b for illustration); or consumer acceptance for RPs is large, but the size of the FOCs segment is intermediate (see Figure 6a for illustration). Ultimately, both the retailer and the new product manufacturer can benefit from 3PR. Similarly, we find that the win–win zone becomes bigger when consumer acceptance for RPs is in certain range, and the size of the FOCs segment is larger than a threshold value by comparing Figure 6a,b. Therefore, when consumer acceptance for RPs is not very high, and the size of the FOCs segment is relatively high, there are more win–win opportunities for the new product manufacturer and retailer.

**Proposition**  **4.** *The retailer’s (new product manufacturer’s) profit is increasing (decreasing) in environmental education* ϕ *. However, the 3PR’s profit will be the highest when both consumer acceptance for RPs and environmental education* ϕ *are in a certain range.*

Instinctively, environmental education has the potential to significantly improve the retailer’s profit as the size of the FOCs segment enlarges, which is unfavorable for the new product manufacturer. For the RPs, according to Lemma 3, the order quantity in model OR is increasing in ϕ. Therefore, when consumer acceptance for RPs is small, the 3PR’s profit will gain as ϕ increases. When consumer acceptance for RPs is large, the 3PR’s profit also increases with the size of the FOCs segment when environmental education is in a certain range. However, when consumer acceptance for RPs and the size of the FOCs segment are both large, the consumers’ types become stable, and consumers have non-differentiated choices for RPs and NPs. At this time, the 3PR’s profit may decrease with environmental education.

## 5. Value of Environmental Education

In this section, we discuss the value of environmental education for the supply chain profit, consumer surplus, and environmental performance.

### 5.1. Value for the Supply Chain

The supply chain profit includes the new product manufacturer’s profit, the 3PR’s profit (model OR), and the retailer’s profit. Therefore, the supply chain’s profit in model OR and model IR are given by

(8) $$SC^{IR}=\pi_{M}^{IR}+E[\pi_{R}^{IR}]$$

(9) $$SC^{OR}=\pi_{M}^{OR}+\pi_{R}^{OR}+\pi_{3PR}$$

**Lemma**  **5.** *The supply chain profits in both model IR and OR (i.e., *$\frac{\partial SC^{IR}}{\partial \phi }>0$  *and* $\frac{\partial SC^{OR}}{\partial \phi }>0$*) are increasing in environmental education. Moreover, the supply chain gains more profits from outsourcing remanufacturing (i.e.,* $SC^{OR}-SC^{IR}>0$).

Lemma 5 shows that the supply chain profit increases with the size of the FOCs segment. A rise in ϕ indicates an increase in the number of consumers with a low willingness can purchase RPs. This is consistent with the conventional wisdom that remanufacturing offers greater value to the supply chain because of greater cost savings. In model OR, the retailer purchases from a 3PR by outsourcing remanufacturing. In this situation, there is no yield uncertainty, and the remanufactured products are not defective. Therefore, the supply chain gains more profits from outsourcing remanufacturing.

### 5.2. Value for the Consumers

In this subsection, we discuss the RPs’ optimal production patterns from consumers’ perspectives and analyze the value of environmental education. Based on our inverse demand functions, consumer surplus is given by

(10) $$CS^{IR}=\int_{1-q_{n}}^{1}\left(v-p_{n}\right)dv+\int_{1-q_{n}-\varepsilon q_{r}}^{1-q_{n}}\left(\delta v-p_{r}\right)dv$$

(11) $$CS^{OR}=\int_{1-q_{n}}^{1}\left(v-p_{n}\right)dv+\int_{1-q_{n}-q_{r}}^{1-q_{n}}\left(\delta v-p_{r}\right)dv$$

**Lemma**  **6.** *Outsourcing remanufacturing benefits the consumer when the retailer’s remanufacturing technology level is low (i.e.,* $x<x_{1}$*).*

Here, $x_{1}=\frac{\delta \phi {\left(1-\delta +\delta \phi \right)}^{2}\left(32-32\delta -4\phi +31\delta \phi \right)}{\left(\delta -1\right)\delta \phi \left(4\left(3\phi -8\right)+\delta \left(64-77\phi +16\phi^{2}\right)+\delta^{2}\left(-32+65\phi -38\phi^{2}+5\phi^{3}\right)\right)}$.

Following Niu et al. [50], we denote $x=\mu^{2}/\sigma^{2}$ as the retailer’s remanufacturing technology level. Lemma 6 shows that outsourcing remanufacturing is more valuable for consumers if the retailer’s remanufacturing technology level is low. When the retailer’s remanufacturing technology level is low, in model OR, it is possible to eliminate yield uncertainty through outsourcing remanufacturing, which in turn can increase the supply competition. Consumers are able to purchase an increased number of NPs and RPs, resulting in an increase in their surpluses. Thus, outsourcing remanufacturing benefits consumers when $x<x_{1}$. On the contrary, in model IR, as a result of the low level of remanufacturing technology at the retailer, the market share of new products expands. Competition between products becomes weaker, resulting in higher prices for new products. Consumer surplus decreases due to lower sales of RPs and higher retail prices for NPs.

### 5.3. Value for the Environment Performance

In this subsection, we investigate the value of environmental education for the environment. This study contains three types of products, NPs, qualified RPs, and defective RPs, where the qualified RPs have the lowest impact among the three products. Consistent with the relevant literature, e.g., Niu et al. [11] and Chen and Chen [51], the environmental impact of qualified RPs is normalized to zero. Then, we use $e_{n}(e_{r})$ to represent the environmental impact of the new product (defective RPs). Therefore, the environmental impact in model OR and model IR are as follows:

(12) $$EI^{IR}=e_{n}q_{n}+e_{r}\left(1-\varepsilon \right)q_{r}$$

(13) $$EI^{OR}=e_{n}q_{n}$$

**Lemma**  **7.** *The environmental impact increases with environmental education. However, the impact of RPs’ production patterns on the environment depends heavily on the environmental impact of defective RPs. The environmental impact in model IR is higher than that in model OR (i.e.,* $EI^{OR}-EI^{IR}<0$*) if* $e_{r}>e_{r}(1)$  *or* $e_{r}(2)<e_{r}<e_{r}(1)$  *and* $\phi <\phi_{6}$*; Otherwise, we have* $EI^{OR}-EI^{IR}>0$*, where* $e_{r}(1)=\frac{\delta \left(\mu^{2}+\sigma^{2}\right)e_{n}}{(4-\delta )(1-\mu )\mu }$  *and* $e_{r}(2)=\frac{\delta \left(\mu^{2}+\sigma^{2}\right)e_{n}}{4(1-\mu )\mu }$.

Lemma 7 sheds light on the relative environmental friendliness of defective RPs and consumer education on the environment. When the environmental impact of defective RPs is large, it means that there are yield uncertainty and higher environmental impact from defective RPs, which has a greater impact on the environment in model IR. When the environmental impact of defective RPs is intermediate, and the size of the FOCs segment is small, it indicates that there are fewer FOCs purchasing the remanufactured products, which reduces the sales of remanufactured products. As a result, the yield uncertainty, reduced sales of RPs, and relatively higher environmental impacts from defective RPs make the environmental impact higher in model IR than that in model OR. Therefore, the relatively higher environmental impact of defective RPs and temperate environmental education can enhance the environmental sustainability of outsourcing remanufacturing.

## 6. Conclusions

This paper investigates how environmental education affects the decision of the remanufactured products’ production patterns when considering environmental education and yield uncertainty. We consider a new product manufacturer and a retailer who procures the new products and also sells the remanufactured products. The retailer has two production patterns for remanufactured products: in-house remanufacturing with yield uncertainty or outsourced remanufacturing from a 3PR without yield uncertainty. The main findings of this paper are as follows.

First, we examine the equilibrium outcomes of wholesale prices and order quantities under in-house remanufacturing and outsourced remanufacturing. This study found that the wholesale prices in the two models are all decreasing in environmental education. Moreover, the wholesale prices of NPs in model OR are higher than that in model IR when both the consumer acceptance for the RPs and yield uncertainty are low. While the order quantities are increasing as the size of the FOC segment enlarges. That is, environmental education benefits the sales growth of NPs and RPs. Furthermore, the order quantities of RPs in model OR are smaller than in model IR, while for the order quantities of NPs, it is the opposite.

Then, this study explores the remanufacturing choice of the retailer and new product manufacturer. When yield uncertainty is relatively large, the retailer has a strong incentive to choose outsourcing remanufacturing from a 3PR in order to eliminate yield uncertainty. When the yield uncertainty is relatively small, and the size of the FOC segment and consumer acceptance for RPs are large, the retailer also tends to purchase from a 3PR. While the new product manufacturer prefers in-house remanufacturing when the yield uncertainty is large or when the yield uncertainty is small, as well as the size of the FOC segment and consumer acceptance for RPs, are large. This study also shows that the manufacturer and retailer will achieve a win–win situation through outsourcing remanufacturing to the third-party remanufacturer when environmental education and consumer acceptance for the remanufactured product are both in a certain range. The 3PR’s profit will be the highest when both consumer acceptance for RPs and environmental education are in a certain range.

Finally, this research analyzes the value of environmental education for supply chain profit, consumer surplus, and environmental performance. The supply chain profit increases with the size of the FOCs segment, and the supply chain gain more profits from outsourcing remanufacturing. Outsourcing remanufacturing benefits the consumer surplus when the retailer’s remanufacturing technology level is low. Relatively higher environmental impacts of defective RPs and temperate environmental education can enhance the environmental sustainability of outsourcing remanufacturing.

This study provides useful insights to the retailer and manufacturer in remanufacturing supply chain development. Several possible directions for future research could be derived from this work. First, our analysis normalizes the production costs of new products and remanufactured products to zero in order to focus on the impact of environmental education on the remanufacturing supply chain; in reality, the production of the new product may be more costly, and it might be promising to investigate how environmental education affects equilibrium outcomes considering the NPs’ production costs. Second, our model assumes that there is no yield uncertainty in outsourcing remanufacturing of 3PR; whether all results will hold under a 3PR with yield uncertainty remains an open question.

## Figures and Tables

**Table 1:** Notations.

Notation	Definition
v	Consumer willingness-to-pay for NPs
δ	Consumer acceptance for RPs
ε	Yield uncertainty in the remanufacturing process
ϕ	Size of the FOC segment
$e_{n}/e_{r}$	Unit environmental impact of NPs or defective RPs
$p_{n}/p_{r}$	Retail price of the NPs or RPs
$\pi_{M}/\;\;\pi_{3PR}/\pi_{R}$	Profit function
Decision Variables	
$q_{n}/q_{r}$	Order quantity for NPs or RPs
$w_{n}/w_{r}$	Wholesale price for NPs or RPs

**Table 2:** Outcomes.

Model IR	Model OR
$w_{n}^{IR}=\frac{(1-\delta )\mu^{2}+(1-\delta +\delta \phi )\sigma^{2}}{2(\mu^{2}+\sigma^{2})(1-\delta +\delta \phi )}$	$w_{n}^{OR}=\frac{2(1-\delta )}{4-4\delta +3\delta \phi }$; $w_{r}=\frac{\delta (1-\delta )}{4-4\delta +3\delta \phi }$
$q_{n}^{IR}=\frac{1}{4}$	$q_{n}^{OR}=\frac{1-\delta +\delta \phi }{4-4\delta +3\delta \phi }$
$q_{r}^{IR}=\frac{\mu \phi }{4(\mu^{2}+\sigma^{2})(1-\delta +\delta \phi )}$	$q_{r}^{OR}=\frac{\phi }{8-8\delta +6\delta \phi }$
$\pi_{M}^{IR}=\frac{(1-\delta )\mu^{2}+(1-\delta +\delta \phi )\sigma^{2}}{8(\mu^{2}+\sigma^{2})(1-\delta +\delta \phi )}$	$\pi_{M}^{OR}=\frac{2(1-\delta )(1-\delta +\delta \phi )}{{\left(4-4\delta +3\delta \phi \right)}^{2}}$
$E\left[\pi_{R}^{IR}\right]=\frac{(1-\delta +\delta \phi )\sigma^{2}+(1-\delta +4\delta \phi )\mu^{2}}{16(\mu^{2}+\sigma^{2})(1-\delta +\delta \phi )}$	$\pi_{R}^{OR}=\frac{\left(1-\delta +\delta \phi )(4-4\delta +9\delta \phi \right)}{4{\left(4-4\delta +3\delta \phi \right)}^{2}}$
	$\pi_{3PR}=\frac{(1-\delta )\delta \phi }{2{\left(4-4\delta +3\delta \phi \right)}^{2}}$

**Table 3:** Definitions.

$\delta_{i}(i=0,1,2,3,4)$	$\phi_{i}(i=1,2,3,4,5)$
$\delta_{0}=4-2\sqrt{3(1+\sigma^{2}/\mu^{2})}$	$\phi_{1}=\left(1-\delta )(\mu^{2}-3\sigma^{2}\right)/3\sigma^{2}\delta$
$\delta_{1}=\left(9\mu^{2}+\sigma^{2}-\sqrt{49\mu^{4}+50\mu^{2}\sigma^{2}+\sigma^{4}}\right)/2\mu^{2}$	$\phi_{2}=\delta \left(1-\delta \right)\left(7\mu^{2}-17\sigma^{2}+\sqrt{49\mu^{4}+50\mu^{2}\sigma^{2}+\sigma^{4}}\right)/18\delta^{2}\sigma^{2}$
$\delta_{2}=\left(7\mu^{2}-17\sigma^{2}\right)/\left(7\mu^{2}+\sigma^{2}\right)$	$\phi_{3}=\left(1-\delta )(7\mu^{2}-17\sigma^{2}\right)/18\sigma^{2}\delta$
$\delta_{3}=\left(23\mu^{2}-\sigma^{2}-\sqrt{289\mu^{4}+290\mu^{2}\sigma^{2}+\sigma^{4}}\right)/6\mu^{2}$	$\phi_{4}=\delta \left(1-\delta \right)\left(17\mu^{2}-55\sigma^{2}+\sqrt{289\mu^{4}+290\mu^{2}\sigma^{2}+\sigma^{4}}\right)/54\delta^{2}\sigma^{2}$
$\delta_{4}=\left(17\mu^{2}-55\sigma^{2}\right)/\left(17\mu^{2}-\sigma^{2}\right)$	$\phi_{5}=\left(1-\delta )(17\mu^{2}-55\sigma^{2}\right)/54\sigma^{2}\delta$

## Data Availability

Not applicable.

## Appendix A

Derivation of Table 2.

In model IR, the retailer’s expected profit is $E\;[\pi_{R}^{IR}]=\delta \mu q_{r}^{IR}-\frac{\delta \left(\sigma^{2}+\mu^{2}\right)\left(1-\delta +\delta \phi \right){\left(q_{r}^{IR}\right)}^{2}}{\phi }+q_{n}^{IR}\left(1-q_{n}^{IR}-2\delta \mu q_{r}^{IR}-w_{n}^{IR}\right)$. According to the backward induction, we can obtain $q_{n}^{IR}(w_{n}^{IR})=\frac{\left(1-\delta \right)\left(1-w_{n}^{IR}\right)\left(\mu^{2}+\sigma^{2}\right)+\delta \phi \left(\sigma^{2}-\left(\mu^{2}+\sigma^{2}\right)w_{n}^{IR}\right)}{2\left(1-\delta \right)\mu^{2}+2\sigma^{2}\left(1-\delta +\delta \phi \right)}$. Substituting $q_{n}^{IR}(w_{n}^{IR})$ into the new product manufacturer’s profit function $\underset{w_{n}^{IR}}{Max}\pi_{M}^{NS}=w_{n}^{IR}\frac{\left(1-\delta \right)\left(1-w_{n}^{IR}\right)\left(\mu^{2}+\sigma^{2}\right)+\delta \phi \left(\sigma^{2}-\left(\mu^{2}+\sigma^{2}\right)w_{n}^{IR}\right)}{2\left(1-\delta \right)\mu^{2}+2\sigma^{2}\left(1-\delta +\delta \phi \right)}$, so we have the optimal wholesale price: $w_{n}^{IR}=\frac{(1-\delta )\mu^{2}+(1-\delta +\delta \phi )\sigma^{2}}{2(\mu^{2}+\sigma^{2})(1-\delta +\delta \phi )}$ based on other equilibriums.

In model OR, the retailer’s profit is $\pi_{R}^{OR}=q_{n}^{OR}\left(1-q_{n}^{OR}-\delta q_{r}^{OR}-w_{n}^{OR}\right)+q_{r}^{OR}\left(-\frac{\left(1-\delta \right)\delta q_{r}^{OR}}{\phi }+\delta \left(1-q_{n}^{OR}-\delta q_{r}^{OR}\right)-w_{r}\right)$. Using the backward induction, it is easy to obtain $q_{n}^{OR}(w_{n}^{OR},w_{r})=\frac{\left(1-\delta )(1-w_{n}^{OR})+\phi (w_{r}-\delta w_{n}^{OR}\right)}{2\left(1-\delta \right)}$. Substituting $q_{n}^{OR}(w_{n}^{OR},w_{r})$ and $q_{r}^{OR}(w_{n}^{OR},w_{r})$ into the profit function of the new product manufacturer and 3PR, they decide the $w_{n}^{OR}$ and $w_{r}$ simultaneously, and thus we can obtain $w_{n}{}^{OR}=\frac{2\left(1-\delta \right)}{4-4\delta +3\delta \phi }$, $w_{r}=\frac{\left(1-\delta \right)\delta }{4-4\delta +3\delta \phi }$, based on other equilibriums.

**Proof**  **of**  **Lemma**  **1.** Taking the first-order conditions with respect to ϕ, we have $\frac{\partial w_{n}^{IR}}{\partial \phi }=\frac{\left(\delta -1\right)\delta \mu^{2}}{2\left(\mu^{2}+\sigma^{2}\right){\left(1-\delta +\delta \phi \right)}^{2}}$, $\frac{\partial w_{n}^{OR}}{\partial \phi }=\frac{6\left(\delta -1\right)\delta }{{\left(4-4\delta +3\delta \phi \right)}^{2}}$ and $\frac{\partial w_{r}^{OR}}{\partial \phi }=\frac{3\left(\delta -1\right)\delta^{2}}{{\left(4-4\delta +3\delta \phi \right)}^{2}}$. Since 0 < δ < 1, the items listed above are all negative. □

**Proof**  **of**  **Lemma**  **2.** Comparing the wholesale prices of NPs, $w_{n}{}^{OR}-w_{n}{}^{IR}=\frac{[(1-\delta )(\mu^{2}-3\sigma^{2})-3\sigma^{2}\delta \phi ]\delta \phi }{2(\mu^{2}+\sigma^{2})(1-\delta +\delta \phi )(4-4\delta +3\delta \phi )}$. We define $\phi_{1}=\frac{\left(1-\delta )(\mu^{2}-3\sigma^{2}\right)}{3\sigma^{2}\delta }$ so that we can prove $w_{n}{}^{OR}-w_{n}{}^{IR}>0$ if $3\sigma^{2}<(1-\delta )\mu^{2}$ or $(1-\delta )\mu^{2}<3\sigma^{2}<\mu^{2}$ and $\phi <\phi_{1}$; otherwise, $w_{n}{}^{OR}-w_{n}{}^{IR}<0$. □

**Proof**  **of**  **Lemma**  **3.** Taking the first-order conditions with respect to ϕ, we have $\frac{\partial q_{r}^{IR}}{\partial \phi }=\frac{\left(1-\delta \right)\mu }{4\left(\mu^{2}+\sigma^{2}\right){\left(1-\delta +\delta \phi \right)}^{2}}$, $\frac{\partial q_{r}^{OR}}{\partial \phi }=\frac{2\left(1-\delta \right)}{{\left(4-4\delta +3\delta \phi \right)}^{2}}$, and $\frac{\partial q_{n}^{OR}}{\partial \phi }=\frac{\delta \left(1-\delta \right)}{{\left(4-4\delta +3\delta \phi \right)}^{2}}$. It is obvious that they are all positive. □

**Proof**  **of**  **Lemma**  **4.** Comparing the order quantities of NPs, we have $q_{n}{}^{OR}-q_{n}{}^{IR}=\frac{\delta \phi }{4(4-4\delta +3\delta \phi )}>0$. Comparing the order quantities of RPs, we have $q_{r}{}^{OR}-q_{r}{}^{IR}=\frac{[\delta (2\mu^{2}+2\sigma^{2}-3\mu )+2(1-\delta )(\mu^{2}+\sigma^{2}-2\mu )]\phi }{4(\mu^{2}+\sigma^{2})(1-\delta +\delta \phi )(4-4\delta +3\delta \phi )}$. Because $\mu^{2}+\sigma^{2}<\mu <\frac{3}{2}\mu <2\mu$, $q_{r}{}^{OR}-q_{r}{}^{IR}<0$; It is easy to receive that $p_{r}{}^{IR}-\left(p_{r}{}^{OR}-w_{r}\right)=\frac{\delta (2\left(1-\delta \right)\left(\mu^{2}+3\sigma^{2}\right)+3\mu^{2}\delta \phi )}{4\left(\mu^{2}+\sigma^{2}\right)\left(4-4\delta +3\delta \phi \right)}$, which is positive. □

**Proof**  **of**  **Proposition**  **1.** By comparing the manufacturer’s profits in different models, we have $\pi_{M}^{OR}-\pi_{M}^{IR}=\frac{\delta \phi \left[\left(1-\delta \right)\mu^{2}\left(8-8\delta +7\delta \phi \right)-\sigma^{2}\left(8-16\delta +17\delta \phi +\delta^{2}\left(8-17\phi +9\phi^{2}\right)\right)\right]}{8\left(\mu^{2}+\sigma^{2}\right)\left(1-\delta +\delta \phi \right){\left(4-4\delta +3\delta \phi \right)}^{2}}$. Define $f_{1}(\phi )=\left(1-\delta \right)\mu^{2}\left(8-8\delta +7\delta \phi \right)-\sigma^{2}\left(8-16\delta +17\delta \phi +\delta^{2}\left(8-17\phi +9\phi^{2}\right)\right)$. Then, we can obtain ${f_{1}}^{\prime }(\phi )=7\left(1-\delta \right)\delta \mu^{2}-\sigma^{2}\left(17\delta +\delta^{2}\left(-17+18\phi \right)\right)$ and ${f_{1}}^{\prime \prime }(\phi )=-18\delta^{2}\sigma^{2}<0$, making it is easy to know that ${f_{1}}^{\prime }(\phi )$ is decreasing in ϕ and ${f_{1}}^{\prime }(0)=\delta \left(1-\delta \right)(7\mu^{2}-17\sigma^{2})$. (1) If $7\mu^{2}-17\sigma^{2}<0$, we have ${f_{1}}^{\prime }(0)<0$, so ${f_{1}}^{\prime }(\phi )<0$, and $f_{1}(\phi )$ is decreasing in ϕ. It is easy to obtain $f_{1}(0)=8{\left(1-\delta \right)}^{2}(\mu^{2}-\sigma^{2})$ and $f_{1}(1)=\mu^{2}\left(1-\delta \right)\left(8-\delta \right)-\sigma^{2}\left(8+\delta \right)$. (i) if $\mu^{2}-\sigma^{2}<0$, we have $f_{1}(0)<0$, thus $f_{1}(\phi )<0$, $\pi_{M}^{OR}-\pi_{M}^{IR}<0$. (ii) if $7\sigma^{2}<7\mu^{2}<17\sigma^{2}$, $f_{1}(0)>0$. We need to discuss $f_{1}(1)$. Define $g_{1}(\delta )=\mu^{2}\left(1-\delta \right)\left(8-\delta \right)-\sigma^{2}\left(8+\delta \right)$. There exists a unique solution to $g_{1}(\delta )=0$, which we denote as $\delta_{1}=\frac{9\mu^{2}+\sigma^{2}-\sqrt{49\mu^{4}+50\mu^{2}\sigma^{2}+\sigma^{4}}}{2\mu^{2}}$. If $\delta <\delta_{1}$, we have $g_{1}(\delta )=f_{1}(1)>0$. Based on $f_{1}(0)>0,f_{1}(1)>0$ and ${f_{1}}^{\prime }(\phi )<0$, we can get $f_{1}(\phi )>0$, $\pi_{M}^{OR}-\pi_{M}^{IR}>0$; if $\delta >\delta_{1}$, $g_{1}(\delta )=f_{1}(1)<0$. Based on $f_{1}(0)>0,f_{1}(1)<0$ and ${f_{1}}^{\prime }(\phi )<0$, we can know that there also exists a unique solution to $f_{1}(\phi )=0$ and denote it as $\phi_{2}=\frac{\delta \left(1-\delta \right)\left(7\mu^{2}-17\sigma^{2}+\sqrt{49\mu^{4}+50\mu^{2}\sigma^{2}+\sigma^{4}}\right)}{18\delta^{2}\sigma^{2}}$. If $\phi <\phi_{2}$, we have $\pi_{M}^{OR}-\pi_{M}^{IR}>0$; otherwise (i.e., $\phi >\phi_{2}$), we have $\pi_{M}^{OR}-\pi_{M}^{IR}<0$. (2) if $7\mu^{2}-17\sigma^{2}>0$, we have ${f_{1}}^{\prime }(0)>0$, and ${f_{1}}^{\prime }(\phi )$ is decreasing in ϕ. ${f_{1}}^{\prime }(1)=\delta (7\mu^{2}+\sigma^{2})(\delta -\frac{7\mu^{2}-17\sigma^{2}}{7\mu^{2}+\sigma^{2}})$. We denote $\delta_{2}=\frac{7\mu^{2}-17\sigma^{2}}{7\mu^{2}+\sigma^{2}}$. (i) if $\delta <\delta_{2}$, we have ${f_{1}}^{\prime }(1)>0$, then ${f_{1}}^{\prime }(\phi )>0$ and $f_{1}(0)>0$, thus $f_{1}(\phi )>0$, we have $\pi_{M}^{OR}-\pi_{M}^{IR}>0$. (ii) if $\delta >\delta_{2}$, we have ${f_{1}}^{\prime }(1)<0$. There exists a unique solution to ${f_{1}}^{\prime }(\phi )=0$, we denote it as $\phi_{3}=\frac{\left(1-\delta )(7\mu^{2}-17\sigma^{2}\right)}{18\sigma^{2}\delta }$. As a result, if $\phi <\phi_{3}$, we have ${f_{1}}^{\prime }(\phi )>0$, $f_{1}(\phi )>0$ and $\pi_{M}^{OR}-\pi_{M}^{IR}>0$. As for $\phi >\phi_{3}$, based on $g_{1}(\delta )=f_{1}(1)$, it is easy to obtain $f_{1}(1)>0$ and $f_{1}(\phi )>0$ if $\delta_{2}<\delta <\delta_{1}$; Similar to the previous derivation, if $\delta >\delta_{1}$, we have $f_{1}(\phi_{2})=0$. Thus, if $\phi_{3}<\phi <\phi_{2}$, we have $\pi_{M}^{OR}-\pi_{M}^{IR}>0$; otherwise (i.e., $\phi >\phi_{2}$), we have $\pi_{M}^{OR}-\pi_{M}^{IR}<0$. □

**Proof**  **of**  **Proposition**  **2.** By comparing the retailer’s profits in different models, we have $\pi_{R}^{OR}-E[\pi_{R}^{IR}]=\frac{\delta \phi \left[\left(\delta -1\right)\mu^{2}\left(20-20\delta +17\delta \phi \right)+\sigma^{2}\left(28-56\delta +55\delta \phi +\delta^{2}\left(28-55\phi +27\phi^{2}\right)\right)\right]}{16\left(\mu^{2}+\sigma^{2}\right)\left(1-\delta +\delta \phi \right){\left(4-4\delta +3\delta \phi \right)}^{2}}$. Define $f_{2}(\phi )=\left(\delta -1\right)\mu^{2}\left(20-20\delta +17\delta \phi \right)+\sigma^{2}\left(28-56\delta +55\delta \phi +\delta^{2}\left(28-55\phi +27\phi^{2}\right)\right)$. Then, we can obtain ${f_{2}}^{\prime }(\phi )=17\left(1-\delta \right)\delta \mu^{2}+\sigma^{2}\left(55\delta +\delta^{2}\left(-55+54\phi \right)\right)$ and ${f_{1}}^{\prime \prime }(\phi )=54\delta^{2}\sigma^{2}>0$, making it is easy to know that ${f_{2}}^{\prime }(\phi )$ is increasing in ϕ and ${f_{2}}^{\prime }(0)=\delta \left(1-\delta \right)(55\mu^{2}-17\sigma^{2})$. (1) If $55\sigma^{2}-17\mu^{2}>0$, we have ${f_{2}}^{\prime }(0)>0$, so ${f_{2}}^{\prime }(\phi )>0$ and $f_{2}(\phi )$ is increasing in ϕ. It is easy to get that $f_{2}(0)=4{\left(1-\delta \right)}^{2}(7\sigma^{2}-5\mu^{2})$ and $f_{2}(1)=\mu^{2}\left(\delta -1\right)\left(20-3\delta \right)+\sigma^{2}\left(28-\delta \right)$. (i) If $7\sigma^{2}-5\mu^{2}<0$, we have $f_{2}(0)>0$, thus $f_{2}(\phi )>0$,$\pi_{R}^{OR}-E[\pi_{R}^{IR}]>0$. (ii) If $\frac{17}{55}\mu^{2}<\sigma^{2}<\frac{5}{7}\mu^{2}$, $f_{2}(0)<0$. We need to discuss $f_{2}(1)$. Define $g_{2}(\delta )=f_{2}(1)=\mu^{2}\left(\delta -1\right)\left(20-3\delta \right)+\sigma^{2}\left(28-\delta \right)$. It is easy to conclude that ${g_{2}}^{\prime }(\delta )=(23-6\delta )\mu^{2}-\sigma^{2}>17\mu^{2}-\sigma^{2}>0$. Thus, we can know that $g_{2}(\delta )$ is increasing in δ. Based on $g_{2}(0)=4\left(7\sigma^{2}-5\mu^{2}\right)<0$ and $g_{2}(1)=27\sigma^{2}>0$, there exists a unique solution to $g_{2}(\delta )=0$, which we denote as $\delta_{3}=\frac{23\mu^{2}-\sigma^{2}-\sqrt{289\mu^{4}+290\mu^{2}\sigma^{2}+\sigma^{4}}}{6\mu^{2}}$. If $\delta <\delta_{3}$, we have $g_{2}(\delta )=f_{2}(1)<0$. Based on $f_{2}(0)<0,f_{2}(1)<0$ and ${f_{2}}^{\prime }(\phi )>0$, we can get $f_{2}(\phi )<0$, $\pi_{R}^{OR}-E[\pi_{R}^{IR}]<0$; if $\delta >\delta_{3}$, $g_{2}(\delta )=f_{2}(1)>0$. Based on $f_{2}(0)<0,f_{2}(1)>0$ and ${f_{2}}^{\prime }(\phi )>0$, we can know that there also exists a unique solution to $f_{2}(\phi )=0$ and denote it as $\phi_{4}=\frac{\delta \left(1-\delta \right)\left(17\mu^{2}-55\sigma^{2}+\sqrt{289\mu^{4}+290\mu^{2}\sigma^{2}+\sigma^{4}}\right)}{54\delta^{2}\sigma^{2}}$. If $\phi <\phi_{4}$, we have $\pi_{R}^{OR}-E[\pi_{R}^{IR}]<0$; otherwise (i.e., $\phi >\phi_{4}$), we have $\pi_{R}^{OR}-E[\pi_{R}^{IR}]>0$. (2) if $55\sigma^{2}-17\mu^{2}<0$, we have ${f_{2}}^{\prime }(0)<0$, and ${f_{2}}^{\prime }(\phi )$ is increasing in ϕ.${f_{2}}^{\prime }(1)=(17\mu^{2}-\sigma^{2})(\delta -\frac{17\mu^{2}-55\sigma^{2}}{17\mu^{2}-\sigma^{2}})$. We denote $\delta_{4}=\frac{17\mu^{2}-55\sigma^{2}}{17\mu^{2}-\sigma^{2}}$. (i) If $\delta <\delta_{4}$, we have $f_{2}\prime (1)<0$, then ${f_{2}}^{\prime }(\phi )<0$ and $f_{2}(0)<0$, thus $f_{2}(\phi )<0$, we have $\pi_{R}^{OR}-E[\pi_{R}^{IR}]<0$. (ii) If $\delta >\delta_{4}$, we have ${f_{2}}^{\prime }(1)>0$. There exists a unique solution to ${f_{2}}^{\prime }(\phi )=0$, we denote it as $\phi_{5}=\frac{\left(1-\delta )(17\mu^{2}-55\sigma^{2}\right)}{54\sigma^{2}\delta }$. As a result, if $\phi <\phi_{5}$, we have ${f_{2}}^{\prime }(\phi )<0$ and $f_{2}(0)<0$, thus $f_{2}(\phi )<0$, $\pi_{R}^{OR}-E[\pi_{R}^{IR}]<0$. As for $\phi >\phi_{5}$, based on $g_{2}(\delta )=f_{2}(1)$, it is easy to get $f_{2}(1)<0$ and $f_{2}(\phi )<0$ if $\delta_{4}<\delta <\delta_{3}$; Similar to the previous derivation, if $\delta_{3}<\delta <1$, we have $f_{2}(\phi_{4})=0$. Thus, if $\phi_{5}<\phi <\phi_{4}$, we have $\pi_{R}^{OR}-E[\pi_{R}^{IR}]<0$; otherwise (i.e., $\phi >\phi_{4}$), we have $\pi_{R}^{OR}-E[\pi_{R}^{IR}]>0$. □

**Proof**  **of**  **Proposition**  **3.** Based on proof of Propositions 1 and 2, it is easy to conclude that the new product manufacturer and retailer will achieve a win–win situation under certain conditions. Note, when $\mu^{2}>\frac{17}{7}\sigma^{2}$, it is easy to show that $\delta_{2}<\delta_{3}$ and $\phi_{3}<\phi_{4}$.□

**Proof**  **of**  **Proposition**  **4.** Taking the first-order conditions with respect to ϕ, we have $\frac{\partial E[\pi_{R}^{IR}]}{\partial \phi }=\frac{3\left(1-\delta \right)\delta \mu^{2}}{16\left(\mu^{2}+\sigma^{2}\right){\left(1-\delta +\delta \phi \right)}^{2}}>0$, $\frac{\partial \pi_{R}^{OR}}{\partial \phi }=\frac{\delta \left(1-\delta \right)\left(28-28\delta +33\delta \phi \right)}{2{\left(4-4\delta +3\delta \phi \right)}^{3}}>0$ $\frac{\partial \pi_{M}^{IR}}{\partial \phi }=\frac{\left(\delta -1\right)\delta \mu^{2}}{8\left(\mu^{2}+\sigma^{2}\right){\left(1-\delta +\delta \phi \right)}^{2}}<0$, $\frac{\partial \pi_{M}^{OR}}{\partial \phi }=\frac{2\delta \left(\delta -1\right)\left(2-2\delta +3\delta \phi \right)}{{\left(4-4\delta +3\delta \phi \right)}^{3}}<0$, and $\frac{\partial \pi_{3PR}}{\partial \phi }=\frac{\delta \left(1-\delta \right)\left(4-4\delta -3\delta \phi \right)}{2{\left(4-4\delta +3\delta \phi \right)}^{3}}=\frac{3\delta^{2}\left(1-\delta \right)}{2{\left(4-4\delta +3\delta \phi \right)}^{3}}(\frac{4-4\delta }{3\delta }-\phi )$. This is easy to obtain (i) if $0<\delta <\frac{4}{7}$, we have $\frac{\partial \pi_{3PR}}{\partial \phi }>0$. (ii) if $\delta >\frac{4}{7}$, $\phi <\frac{4-4\delta }{3\delta }$, we have $\frac{\partial \pi_{3PR}}{\partial \phi }>0$; $\phi >\frac{4-4\delta }{3\delta }$, and we have $\frac{\partial \pi_{3PR}}{\partial \phi }<0$. □

**Proof**  **of**  **Lemma**  **5.** According to the definition of supply chain’s profit, we have $SC^{IR}=\frac{(3-3\delta +4\delta \phi )\mu^{2}+3(1-\delta +\delta \phi )\sigma^{2}}{16(\mu^{2}+\sigma^{2})(1-\delta +\delta \phi )}$. Taking the first-order conditions, we have $\frac{\partial SC^{IR}}{\partial \phi }=\frac{\left(1-\delta \right)\delta \mu^{2}}{16\left(\mu^{2}+\sigma^{2}\right){\left(1-\delta +\delta \phi \right)}^{2}}>0$. Similarly, we have $SC^{OR}=\frac{12+\delta \left(23\phi -24\right)+\delta^{2}\left(12-23\phi +9\phi^{2}\right)}{4{\left(4-4\delta +3\delta \phi \right)}^{2}}$ and $\frac{\partial SC^{OR}}{\partial \phi }=\frac{\delta \left(1-\delta \right)\left(20-20\delta +3\delta \phi \right)}{4{\left(4-4\delta +3\delta \phi \right)}^{3}}>0$. Comparing the supply chain’s profits in different models, we have $SC^{OR}-SC^{IR}=\frac{\delta \phi \left[\left(1-\delta \right)\mu^{2}\left(4-4\delta +5\delta \phi \right)+\sigma^{2}\left(20-40\delta +29\delta \phi +\delta^{2}\left(20-29\phi +9\phi^{2}\right)\right)\right]}{16\left(\mu^{2}+\sigma^{2}\right)\left(1-\delta +\delta \phi \right){\left(4-4\delta +3\delta \phi \right)}^{2}}$. Define $f_{3}(\phi )=\left(1-\delta \right)\mu^{2}\left(4-4\delta +5\delta \phi \right)+\sigma^{2}\left(20-40\delta +29\delta \phi +\delta^{2}\left(20-29\phi +9\phi^{2}\right)\right)$. Then, we can obtain ${f_{3}}^{\prime }(\phi )=5\left(1-\delta \right)\delta \mu^{2}+29\delta \sigma^{2}(1-\delta )+18\delta^{2}\sigma^{2}\phi >0$, making it is easy to know that $f_{3}(\phi )$ is increasing in ϕ and $f_{3}(0)=4{\left(1-\delta \right)}^{2}(\mu^{2}+5\sigma^{2})>0$. Based on the above discussion, we have $f_{3}(\phi )>0$ and $SC^{OR}-SC^{IR}>0$. □

**Proof**  **of**  **Lemma**  **6.** By comparing the consumer surplus in different models, we have $CS^{OR}-E[CS^{IR}]=\frac{A\left(\delta \right)-B\left(\delta \right)x}{32\left(1+x\right){\left(1-\delta +\delta \phi \right)}^{2}{\left(4-4\delta +3\delta \phi \right)}^{2}}$, where $x=\frac{\mu^{2}}{\sigma^{2}}$, $A\left(\delta \right)=\delta \phi {\left(1-\delta +\delta \phi \right)}^{2}\left(32-32\delta -4\phi +31\delta \phi \right)$ and $B\left(\delta \right)=\left(\delta -1\right)\delta \phi \left(4\left(3\phi -8\right)+\delta \left(64-77\phi +16\phi^{2}\right)+\delta^{2}\left(-32+65\phi -38\phi^{2}+5\phi^{3}\right)\right)$. The item 32−32δ−4ϕ+31δϕ=(32−4ϕ)(1−δ)+27δϕ>0 makes it is easy to know that A(δ)>0. Define $B_{1}\left(\delta \right)=4\left(3\phi -8\right)+\delta \left(64-77\phi +16\phi^{2}\right)+\delta^{2}\left(-32+65\phi -38\phi^{2}+5\phi^{3}\right)$, and we can obtain ${B_{1}}^{\prime }\left(\delta \right)=64-77\phi +16\phi^{2}+2\delta \left(-32+65\phi -38\phi^{2}+5\phi^{3}\right)$ and ${B_{1}}^{\prime \prime }\left(\delta \right)=2\left(\phi -1\right)\left(5\phi^{2}-33\phi +32\right)<0$. Then, we have ${B_{1}}^{\prime }\left(0\right)=64-77\phi +16\phi^{2}>0,{B_{1}}^{\prime }\left(1\right)=\phi \left(53-60\phi +10\phi^{2}\right)>0,{B_{1}}^{\prime }\left(\delta \right)>0$, $B_{1}\left(0\right)=4\left(3\phi -8\right)<0$ and $B_{1}\left(1\right)=\phi^{2}\left(5\phi -22\right)<0$. Thus, we can obtain $B_{1}\left(\delta \right)<0$ and B(δ)>0. As a result, if $x<x_{1}$ we have $CS^{oR}-E[CS^{IR}]>0$; if $x>x_{1}$ we have $CS^{oR}-E[CS^{IR}]<0$. Here, $x_{1}=\frac{A}{B}=\frac{\delta \phi {\left(1-\delta +\delta \phi \right)}^{2}\left(32-32\delta -4\phi +31\delta \phi \right)}{\left(\delta -1\right)\delta \phi \left(4\left(3\phi -8\right)+\delta \left(64-77\phi +16\phi^{2}\right)+\delta^{2}\left(-32+65\phi -38\phi^{2}+5\phi^{3}\right)\right)}$. □

**Proof**  **of**  **Lemma**  **7.** According to the expression of environmental performance, we have $EI^{IR}=\frac{e_{n}}{4}+\frac{\left(1-\mu \right)\mu \phi e_{r}}{4\left(\mu^{2}+\sigma^{2}\right)\left(1-\delta +\delta \phi \right)}$ and $EI^{OR}=\frac{\left(1-\delta +\delta \phi \right)e_{n}}{4-4\delta +3\delta \phi }$. Taking the first-order conditions with respect to ϕ, we have $\frac{\partial EI^{IR}}{\partial \phi }=\frac{\left(1-\delta \right)\left(1-\mu \right)\mu e_{r}}{4\left(\mu^{2}+\sigma^{2}\right){\left(1-\delta +\delta \phi \right)}^{2}}>0$ and $\frac{\partial EI^{OR}}{\partial \phi }=\frac{\left(1-\delta \right)\delta e_{n}}{{\left(4-4\delta +3\delta \phi \right)}^{2}}>0$. By comparing the environmental performance, we have $EI^{OR}-EI^{IR}=\frac{\phi \left(1-\delta \right)\left[\delta \left(\mu^{2}+\sigma^{2}\right)e_{n}-4\left(1-\mu \right)\mu e_{r}\right]+\delta \phi^{2}\left[\delta \left(\mu^{2}+\sigma^{2}\right)e_{n}-3\left(1-\mu \right)\mu e_{r}\right]}{4\left(\mu^{2}+\sigma^{2}\right)\left(1-\delta +\delta \phi \right)\left(4-4\delta +3\delta \phi \right)}$. Define $e_{r}(0)=\frac{\delta \left(\mu^{2}+\sigma^{2}\right)e_{n}}{3(1-\mu )\mu }$, $e_{r}(1)=\frac{\delta \left(\mu^{2}+\sigma^{2}\right)e_{n}}{(4-\delta )(1-\mu )\mu }$, $e_{r}(2)=\frac{\delta \left(\mu^{2}+\sigma^{2}\right)e_{n}}{4(1-\mu )\mu }$ and $\phi_{6}=\frac{4\left(1-\delta \right)\left(e_{r}-e_{r}(2)\right)}{3\delta \left(e_{r}(0)-e_{r}\right)}$. (1) if $e_{r}\leq e_{r}(2)$, we have $EI^{OR}-EI^{IR}>0$; (2) if $e_{r}\geq e_{r}(0)$, we have $EI^{OR}-EI^{IR}<0$; (3) if $e_{r}(1)<e_{r}<e_{r}(0)$, we have $EI^{OR}-EI^{IR}<0$; (4) if $e_{r}(2)<e_{r}<e_{r}(1)$, $\phi <\phi_{6}$, we have $EI^{OR}-EI^{IR}<0$; $\phi >\phi_{6}$, we have $EI^{OR}-EI^{IR}>0$. In summary, (1) if $e_{r}\leq e_{r}(2)$, $EI^{OR}-EI^{IR}>0$; (2) if $e_{r}\geq e_{r}(1)$, we have $EI^{OR}-EI^{IR}<0$; (3) if $e_{r}(2)<e_{r}<e_{r}(1)$, $\phi <\phi_{6}$, we have $EI^{OR}-EI^{IR}<0$ or $\phi >\phi_{6}$, $EI^{OR}-EI^{IR}>0$. □

## References

[1] Aydin R., Mansour M. (2023). Investigating sustainable consumer preferences for remanufactured electronic products. J. Eng. Res..

[2] Forti V., Baldé C., Kuehr R., Bel G. (2020). The Global E-waste Monitor 2020. Quantities, Flows, and the Circular Economy Potential.

[3] Ginsburg J. (2001). Manufacturing: Once is not enough. BusinessWeek.

[4] Vasudevan H., Kalamkar V., Terkar R. (2012). Remanufacturing for sustainable development: Key challenges, elements, and benefits. Int. J. Innov. Manag. Technol..

[5] Khor K.S., Hazen B.T. (2016). Remanufactured products purchase intentions and behaviour: Evidence from Malaysia. Int. J. Prod. Res..

[6] Zsoka A., Szerenyi Z.M., Szechy A., Kocsis T. (2013). Greening due to environmental education? Environmental knowledge, attitudes, consumer behavior and everyday pro-environmental activities of Hungarian high school and university students. J. Clean. Prod..

[7] Zhang A., Venkatesh V.G., Liu Y., Wan M., Qu T., Huisingh D. (2019). Barriers to smart waste management for a circular economy in China. J. Clean. Prod..

[8] Qu Y., Liu Y., Guo L., Zhu Q., Tseng M. (2018). Promoting remanufactured heavy-truck engine purchase in China: Influencing factors and their effects. J. Clean. Prod..

[9] Li Y.K., Chen J., Sohail M.T. (2022). Does education matter in China? Myths about financial inclusion and energy consumption. Environ. Sci. Pollut. Res..

[10] Yang T.J., Li C.M., Yue X.P., Zhang B.B. (2022). Decisions for Blockchain Adoption and Information Sharing in a Low Carbon Supply Chain. Mathematics.

[11] Niu B., Bao J., Cao B. (2022). Retailer’s make-or-buy decision for remanufactured products under in-house yield uncertainty. Omega.

[12] Alyahya M., Agag G., Aliedan M., Abdelmoety Z.H., Daher M.M. (2023). A sustainable step forward: Understanding factors affecting customers’ behaviour to purchase remanufactured products. J. Retail. Consum. Serv..

[13] Dobbelstein T., Lochner C. (2023). Factors influencing purchase intention for recycled products: A comparative analysis of Germany and South Africa. Sustain. Dev..

[14] Alqahtani A.Y., Gupta S.M. (2017). Warranty as a marketing strategy for remanufactured products. J. Clean. Prod..

[15] Yan W., Xiong Y., Xiong Z., Guo N. (2015). Bricks vs. clicks: Which is better for marketing remanufactured products?. Eur. J. Oper. Res..

[16] Xue D., Teunter R.H., Zhu S.X., Zhou W. (2021). Entering the high-end market by collecting and remanufacturing a competitor’s high-end cores. Omega.

[17] Alegoz M. (2022). Simultaneous remanufacturing and government incentives in remanufacturing systems. Eur. J. Ind. Eng..

[18] Xia Y., Tan D., Wang B. (2021). Use of a product service system in a competing remanufacturing market. Omega.

[19] Jin M., Nie J., Yang F., Zhou Y. (2017). The impact of third-party remanufacturing on the forward supply chain: A blessing or a curse?. Int. J. Prod. Res..

[20] Huang H., Meng Q., Xu H., Zhou Y. (2019). Cost information sharing under competition in remanufacturing. Int. J. Prod. Res..

[21] Baghdadi E., Shafiee M., Alkali B. (2022). Upgrading Strategy, Warranty Policy and Pricing Decisions for Remanufactured Products Sold with Two-Dimensional Warranty. Sustainability.

[22] Qiao H., Su Q. (2020). The prices and quality of new and remanufactured products in a new market segment. Int. Trans. Oper. Res..

[23] Hsieh C.-C., Lai H.-H. (2020). Pricing and ordering decisions in a supply chain with downward substitution and imperfect process yield. Omega.

[24] Hsu A., Bassok Y. (1999). Random yield and random demand in a production system with downward substitution. Oper. Res..

[25] Metzker P., Thevenin S., Adulyasak Y., Dolgui A. (2023). Robust optimization for lot-sizing problems under yield uncertainty. Comput. Oper. Res..

[26] Freeman N.K., Narayanan A., Keskin B.B. (2021). Optimal use of downward substitution in a manufacturing operation subject to uncertainty. Omega.

[27] Peng Y., Yan X.M., Zhou A., Cheng T.C.E., Ji M. (2023). Entry Game in Supply Chains with Yield Uncertainty. J. Ind. Manag. Optim..

[28] Sharma B.P., Yu T.E., English B.C., Boyer C.N., Larson J.A. Stochastic optimization of cellulosic biofuel supply chain incorporating feedstock yield uncertainty. Proceedings of the 10th International Conference on Applied Energy (ICAE).

[29] Cai J.H., Sun H.N., Li X.J., Ergu D.J. (2022). Input Quantity Decision and Coordination Mechanism with Two Production Chances and Yield Uncertainty. Int. J. Inf. Technol. Decis. Mak..

[30] Cai J.H., Sun H.N., Wei B. (2022). Supply chain coordination with strategic customers: Yield uncertainty and replenishment tactic. J. Oper. Res. Soc..

[31] Kouvelis P., Li J. (2013). Offshore Outsourcing, Yield Uncertainty, and Contingency Responses. Prod. Oper. Manag..

[32] Yuan X., Bi G., Fei Y., Liu L. (2021). Supply chain with random yield and financing. Omega.

[33] Talay I., Ozdemir-Akyildirim O. (2019). Optimal procurement and production planning for multi-product multi-stage production under yield uncertainty. Eur. J. Oper. Res..

[34] Shao L.S., Wang D.R., Wu X.L. (2022). Competitive trading in forward and spot markets under yield uncertainty. Prod. Oper. Manag..

[35] Lowe J.J., Khademi A., Mason S.J., IEEE Robust Semiconductor Production Planning under Yield Uncertainty. Proceedings of the Winter Simulation Conference (WSC).

[36] Liao H., Zhang Q., Shen N., Nie Y., Li L. (2021). Coordination between forward and reverse production streams for maximum profitability. Omega.

[37] Zhu X., Wang M., Pei J., Pardalos P.M. (2020). Investigating remanufacturing competition with yield uncertainty on market share, profit, and consumer surplus. Int. T. Oper. Res..

[38] Begum A., Liu J.W., Qayum H., Mamdouh A. (2022). Environmental and Moral Education for Effective Environmentalism: An Ideological and Philosophical Approach. Int. J. Environ. Res. Public Health.

[39] Yang B., Wu N.N., Tong Z.P., Sun Y. (2022). Narrative-Based Environmental Education Improves Environmental Awareness and Environmental Attitudes in Children Aged 6–8. Int. J. Environ. Res. Public Health.

[40] Niu Y.F., Wang X., Lin C.Y. (2022). A Study on the Impact of Organizing Environmental Awareness and Education on the Performance of Environmental Governance in China. Int. J. Environ. Res. Public Health.

[41] van de Wetering J., Leijten P., Spitzer J., Thomaes S. (2022). Does environmental education benefit environmental outcomes in children and adolescents? A meta-analysis. J. Environ. Psychol..

[42] Steils N. (2021). Using in-store customer education to act upon the negative effects of impulsiveness in relation to unhealthy food consumption. J. Retail. Consum. Serv..

[43] Lieflander A.K., Frohlich G., Bogner F.X., Schultz P.W. (2013). Promoting connectedness with nature through environmental education. Environ. Educ. Res..

[44] Agrawal V.V., Ferguson M., Toktay L.B., Thomas V.M. (2012). Is Leasing Greener than Selling?. Manag. Sci..

[45] Merkuryev Y., Bikovska J., Merkuryeva G. SUPPLY CHAIN DYNAMICS: SIMULATION-BASED TRAINING AND EDUCATION. Proceedings of the 13th International Conference on Harbor, Maritime and Multimodal Logistics Modeling and Simulation (HMS).

[46] Zhong L., Nie J., Lim M.K., Xia S. (2021). Agency or Self-Run: The effect of consumer green education on recyclers’ distribution channel choice under platform economy. Int. J. Logist. Res. Appl..

[47] Zhou Y., Xiong Y., Jin M. (2021). Less is more: Consumer education in a closed-loop supply chain with remanufacturing. Omega.

[48] Wang M., Yang F., Xia Q. (2021). Design of the reverse channel for the third-party remanufacturing considering consumer education. RAIRO—Oper. Res..

[49] Wang M., Ang S., Yang F., Song J. (2022). Warranty or education?: An analysis of marketing strategy choices for remanufactured products. Asia Pac. J. Mark. Logist..

[50] Niu B., Li J., Zhang J., Cheng H.K., Tan Y.R. (2019). Strategic Analysis of Dual Sourcing and Dual Channel with an Unreliable Alternative Supplier. Prod. Oper. Manag..

[51] Chen Y., Chen F. (2019). On the Competition between Two Modes of Product Recovery: Remanufacturing and Refurbishing. Prod. Oper. Manag..

